# Supplementary material for: Widespread reorganisation of pluripotent factor binding and gene regulatory interactions between human pluripotent states
Source: Nat Commun. 2021 Apr 7;12:2098. doi: 10.1038/s41467-021-22201-4 (PMC8026613; doi:10.1038/s41467-021-22201-4)
Supplement: Supplementary file 1 — Supplementary Information [file 41467_2021_22201_MOESM1_ESM.pdf]

Supplementary information

Widespread reorganisation of pluripotent factor binding and gene regulatory interactions between human pluripotent states

Chovanec, Collier et al.

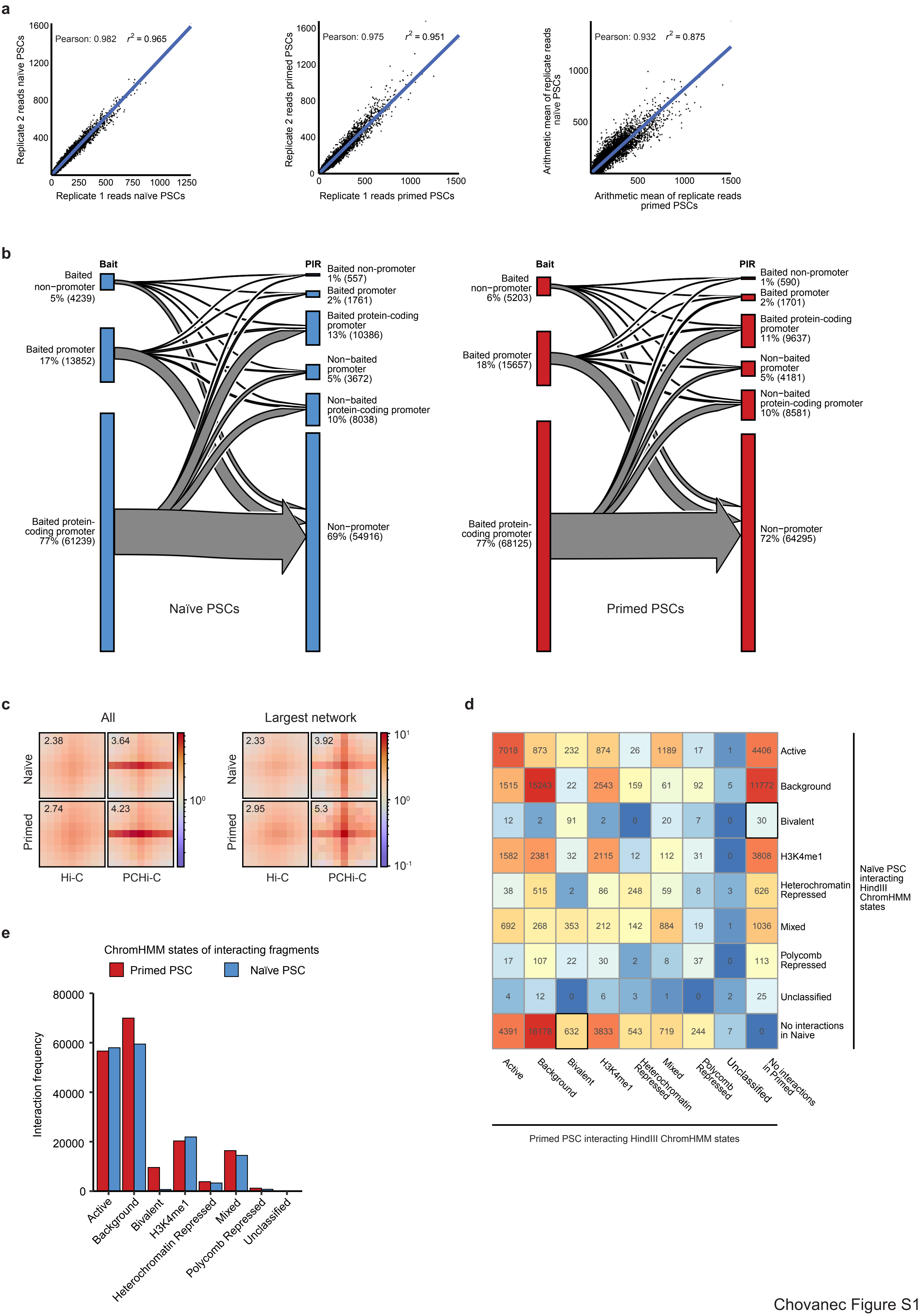

Chovanec Figure S1

Supplementary Fig. 1: Characterisation of PCHi-C replicates and interacting regions

a Scatter plots show pairwise comparisons between individual PCHi-C replicates (naive, left; primed, centre) and between the mean of replicate reads in naive and primed PSCs (right). Correlation values are shown.

b Sankey plots describe the genomic features of interacting regions in naive PSCs (left) and primed PSCs (right) that were detected by PCHi-C. Listed within the 'bait' category are the number and percentage of protein-coding promoters, non-protein-coding promoters and non-promoter regions. The 'PIR' category describes the promoter-interacting regions. As expected, the most frequent type of interaction in both cell types was between baited protein-coding promoters and non-promoter regions.

c Hi-C and PCHi-C data (without significance filtering) were binned at a 25kb resolution and normalised using ICE. Coordinates of all HindIII fragments (left) with a significant PCHi-C interaction (naive, n=47,598; primed, n=50,591) and (right) within the largest sub-network (naive, n=1,035; primed, n=1,611) were used to extract all Hi-C and PCHi-C overlapping bins from which the pileup figures were constructed using coolpuppy[1]. Each pixel on the pileup plots is 25kb with 100kb padding around the central pixel. The change of color shows enrichment of interactions on a log-scale. The value of the central pixel is shown in the top left.

d Heatmap shows the number of interacting HindIII fragments that were assigned to each of the ChromHMM-defined chromatin states in both cell types. Note the higher number of bivalently-marked (H3K27me3 and H3K4me1/3) interacting regions in primed compared to naive PSCs.

e Chart shows the total number of interacting HindIII fragments for each of the ChromHMM states.

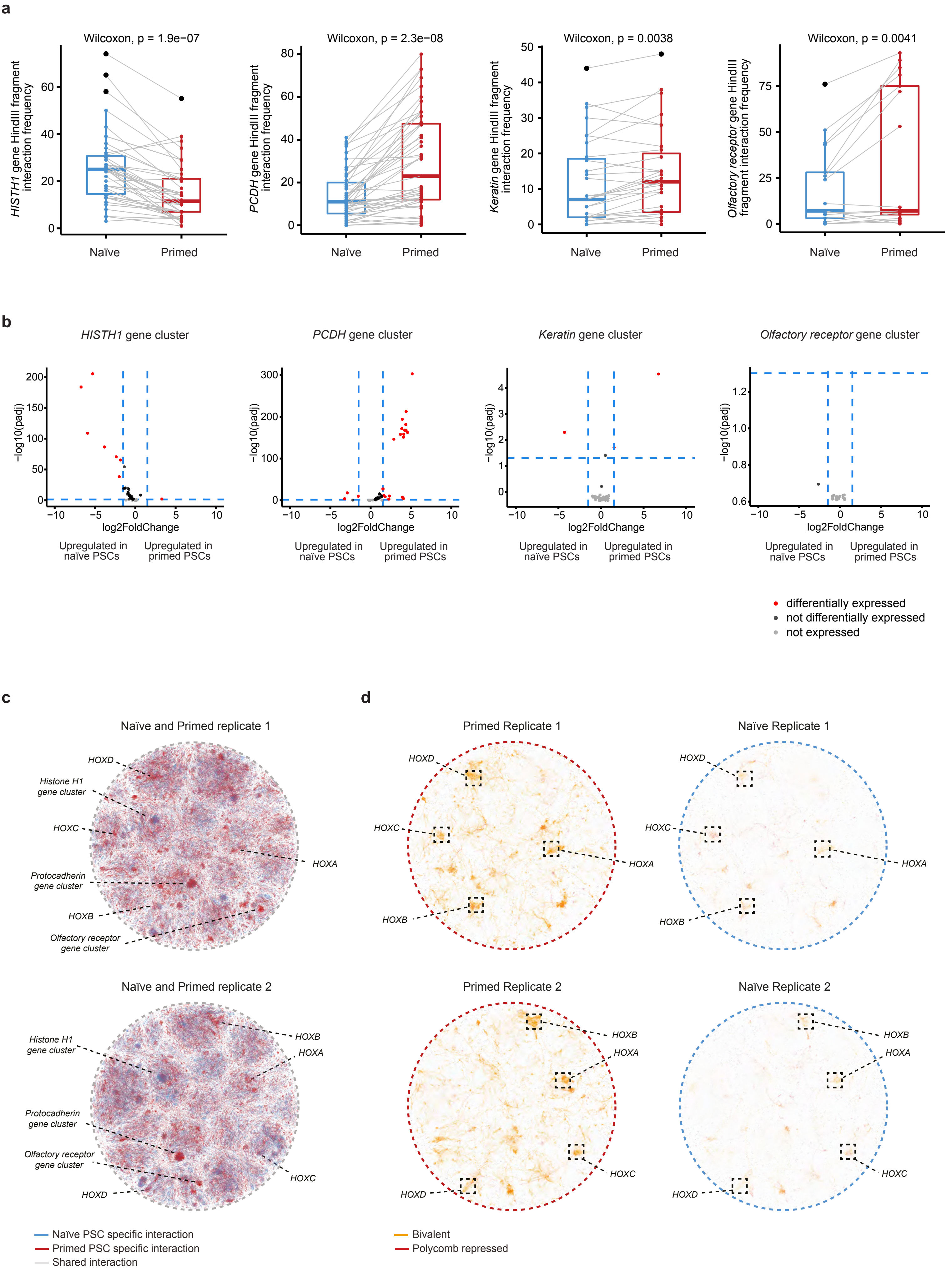

Chovanec Figure S2

Supplementary Fig. 2: Changes in promoter-interaction frequency and transcriptional levels in four gene clusters

a Box plots show the number of significant interactions at HindIII fragments located within the *HISTH1* (n=42), *PCDH* (n=47), *KRT* (n=31) and Olfactory receptor (n=17) gene clusters. The box bounds the interquartile range (IQR) divided by the median (horizontal line), and Tukey-style whiskers extend to a maximum of 1.5 × IQR beyond the box. Blue and red circles indicate individual data points and the black circles indicate individual outliers. The grey lines connect the data points for the same HindIII fragment in the two cell types.

b Volcano plots show the transcriptional changes between naive and primed PSCs for genes within the *HISTH1*, *PCDH*, *KRT* and Olfactory receptor gene clusters. Each dot represents a different gene. Genes coloured in red are differentially expressed between naive and primed PSCs (log10 fold change >1.5 or > -1.5 and with an adjusted *P*-value < 0.05). Other categories shown include genes that are not differentially expressed (log10 fold change <1.5 < -1.5 and/or with an adjusted *P*-value > 0.05) or genes that are not expressed (0 read counts in RNA-seq data).

c Network graphs visualise data from PCHi-C replicates 1 (upper) or replicates 2 (lower) separately. The key landmarks of the networks are indicated (based on Fig. 1b) and are consistent in both network graphs, which provides support for the good reproducibility between replicate samples.

d Related to Fig. 3c, network graphs based on individual PCHi-C replicate samples show the reproducibility of Polycomb-associated interaction clusters in primed and naive PSCs.

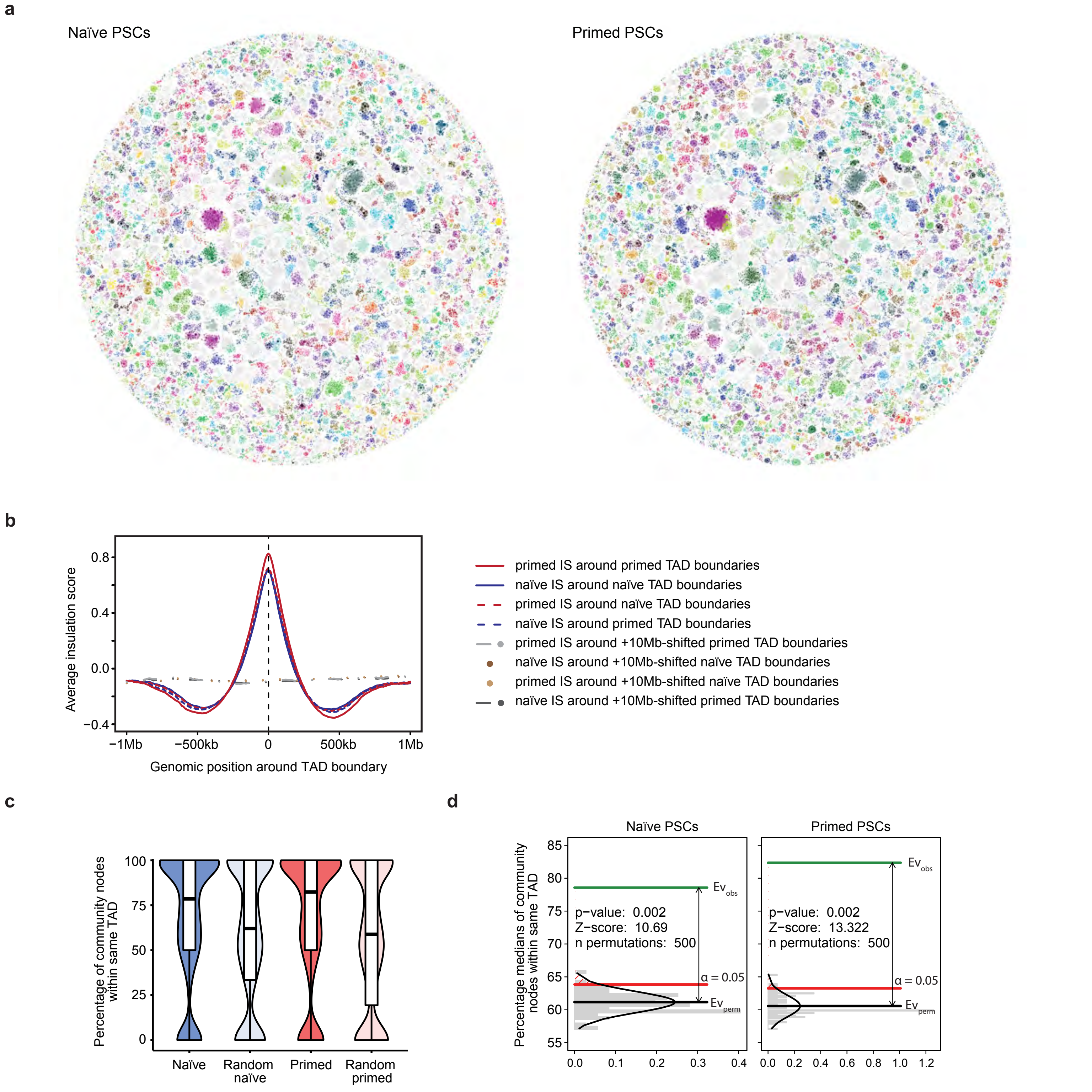

Chovanec Figure S3

Supplementary Fig. 3: Identified communities recapitulate TADs and are largely shared between naive and primed PSCs.

a TADs were defined using matched Hi-C data and their locations are projected onto the PCHi-C network graph. Each TAD is represented by a unique colour and TADs shared between naive and primed PSCs have the same colour. Nodes that fall outside of a defined TAD are coloured in grey.

b Insulation score (IS) profiles around TAD boundaries in naive and primed PSCs. The dotted lines show the IS around boundaries that have been computationally shifted by +1 Mb.

c Plot (left) shows the percentage of nodes within each community that are contained within the same TAD. For both cell types, the percentage was significantly higher compared to a set of randomly shuffled TAD coordinates. The inner box bounds the IQR divided by the median (horizontal line), and Spear-style whiskers extend to the minimum and maximum of the data values.

d Statistical test for (**c**). Bar chart shows the frequency of median percentages obtained after 500 random permutations of TAD coordinates (grey) and the comparison between this control set (Ev_{perm}) and the observed values (Ev_{obs}) using a permutation test (n=500) within the regioneR package (Gel et al. 2016).

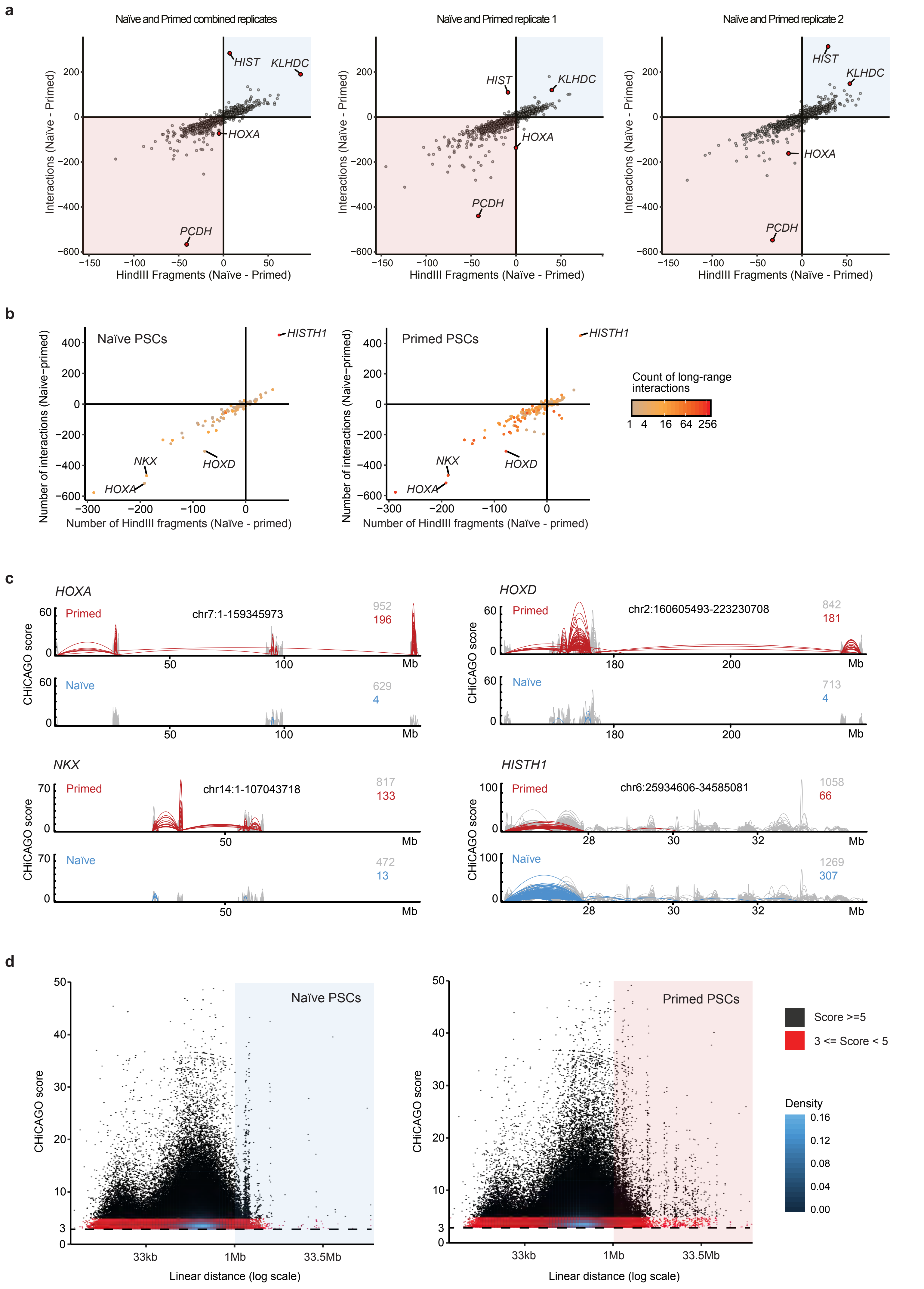

Chovanec Figure S4

Supplementary Fig. 4: Long-range promoter interactions create large sub-networks in primed PSCs

a Scatter plots show the number of interactions (edges) and the number of interacting HindIII fragments (nodes) for each cluster, defined as sub-networks or communities within a sub-network. Sub-networks with a modularity score of 0.7 or above were split into individual communities to ensure interaction clusters were not biased between the two replicates by outlier interactions. The *HOXA*, *HIST1*, *PCDH*, and *KLHDC* clusters are highlighted.

b Scatter plots show the number of interactions (edges) and the number of interacting HindIII fragments (nodes) for each sub-network in naive and primed PSCs. The lower-left quadrant contains larger sub-networks in primed PSCs, and the upper-right quadrant contains larger sub-networks in naive PSCs. The *HOXA*, *HOXD*, *NKX* and *HISTH1* sub-networks are highlighted. Sub-networks are coloured according to their number of long-range promoter interactions. Note the increased number of long-range promoter interactions within most sub-networks in primed (right) compared to naive (left) PSCs.

c Genome browser tracks show the PCHi-C interactions and CHiCAGO scores in naive and primed PSCs for the *HOXA*, *HOXD*, *NKX* and *HISTH1* sub-networks.

d Dot plots show that the high number of long-range promoter interactions in primed PSCs is independent of the applied CHiCAGO threshold. Each dot represents a PCHi-C interaction, positioned according to the linear genomic distance of the interaction (x-axis) and the assigned CHiCAGO score (y-axis). Black dots show the interactions obtained when applying a CHiCAGO score of >5 (the threshold used for constructing the network graph) and red dots show the interactions when using a relaxed CHiCAGO score of between 3 and 5. Primed PSCs have more long-range promoter interactions (shaded area; defined as >1Mb) compared to naive PSCs when either CHiCAGO threshold score is applied.

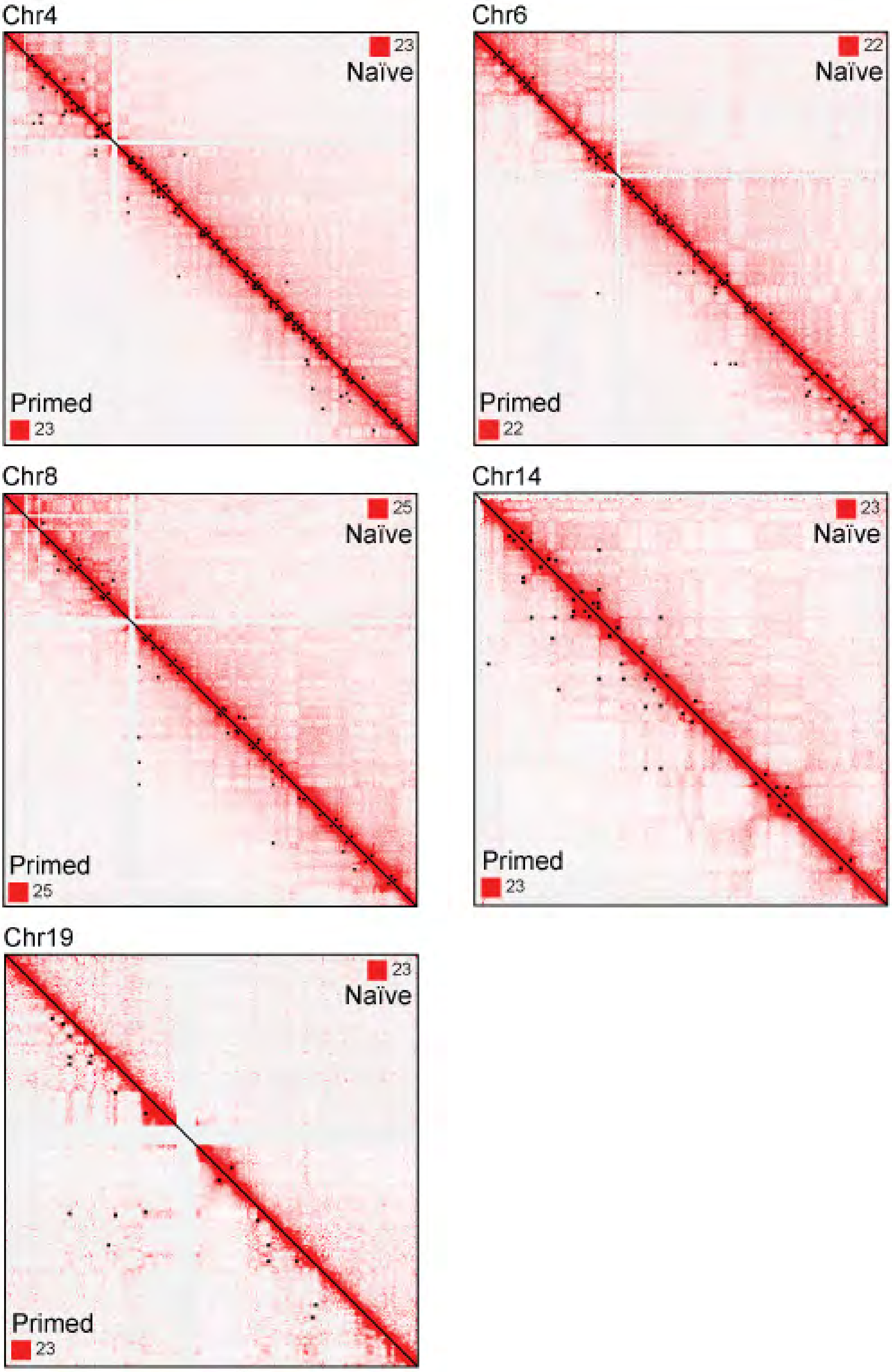

Chovanec Figure S5

Supplementary Fig. 5: Additional examples show the higher number of long-range chromatin interactions in primed compared to naive PSCs

Five examples from different chromosomes of Hi-C interaction matrices at a resolution of 250 kb with Knight-Ruiz (KR) normalisation. In each contact matrix, naive PSCs are shown in the upper right and primed PSCs are shown in the lower left. Areas of contact enrichment were defined separately for naive and primed PSCs using HiCCUPS analysis of Hi-C data and each cell type-specific set of chromatin interactions are highlighted as a black square on their respective heatmaps. The numbers in each corner indicate the maximum intensity values for the matrix.

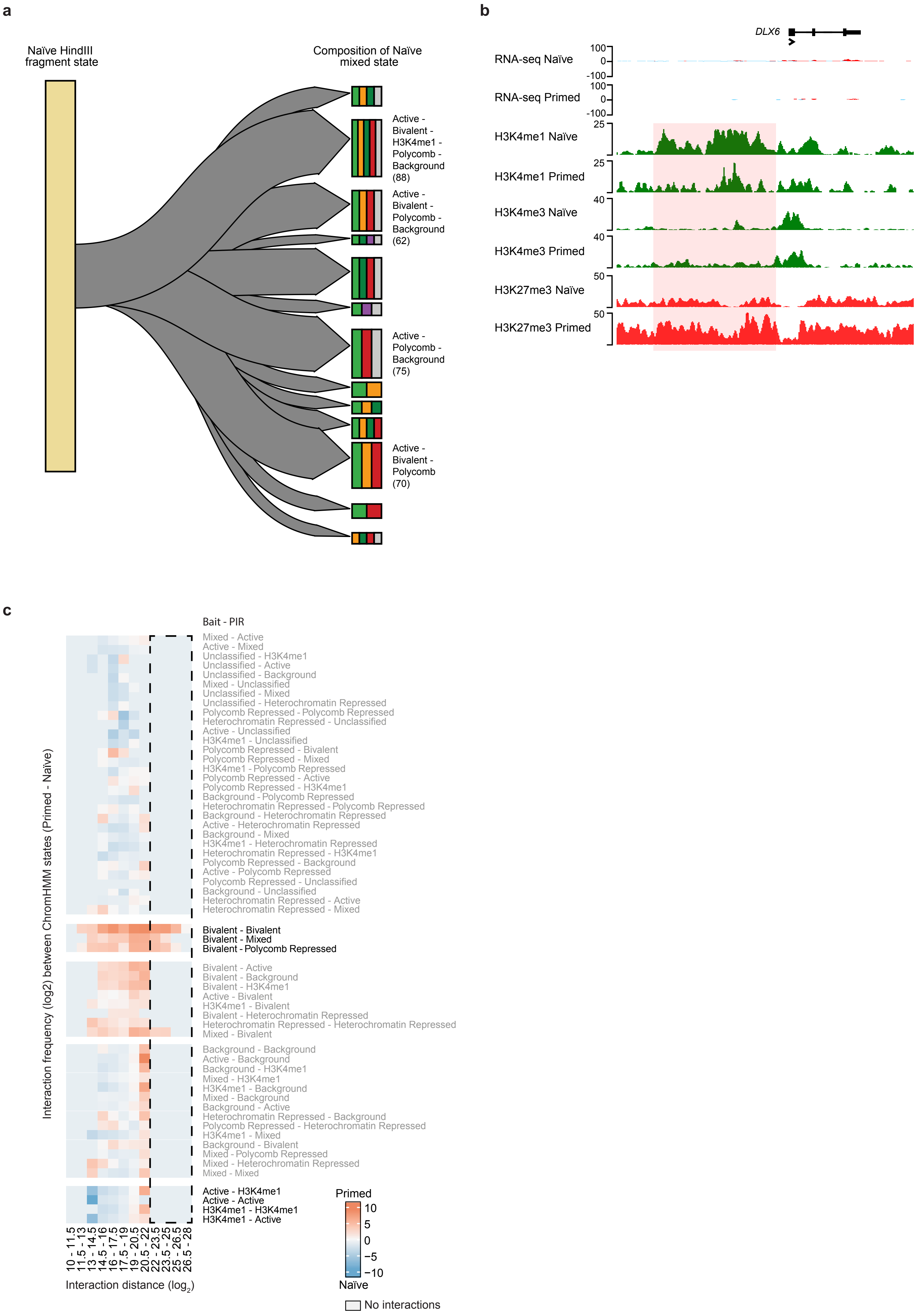

Chovanec Figure S6

Supplementary Fig. 6: Assigning ChromHMM states to chromatin interacting regions

a Sankey plot reveals the chromatin state composition for the 617 HindIII interacting regions in naive PSCs that were defined by ChromHMM as being in a 'mixed' chromatin state. The left column shows the 617 mixed chromatin state regions and the right column shows the breakdown of individual chromatin states for each of the regions, represented by the different colour bars. Approximately half of the mixed state HindIII fragments contain signatures of active (H3K4me3, green), bivalent (H3K27me3 and H3K4me1/3, orange) and Polycomb (H3K27me3-only, red) chromatin.

b Genome browser representation shows an example of a developmental gene marked by active histone modifications in naive PSCs. The *DLX6* upstream region (shaded in red box) is decorated with high H3K4me1 and low H3K27me3 in naive PSCs, and the H3K27me3 signal is much higher across this region in primed PSCs. *DLX6* is transcriptionally inactive in naive and primed PSCs.

c Heatmap shows the difference in HindIII fragment interaction frequency between cell types as a function of the chromatin state of the interacting regions (rows) and the linear interaction distance (columns, binned distances). Interacting regions that are engaged in long-range promoter interactions, defined as >1Mb, are highlighted by the dashed box. Nearly all (98%) of the long-range interactions were associated with bivalently-marked promoters and these regions have a higher interaction frequency in primed compared to naive PSCs.

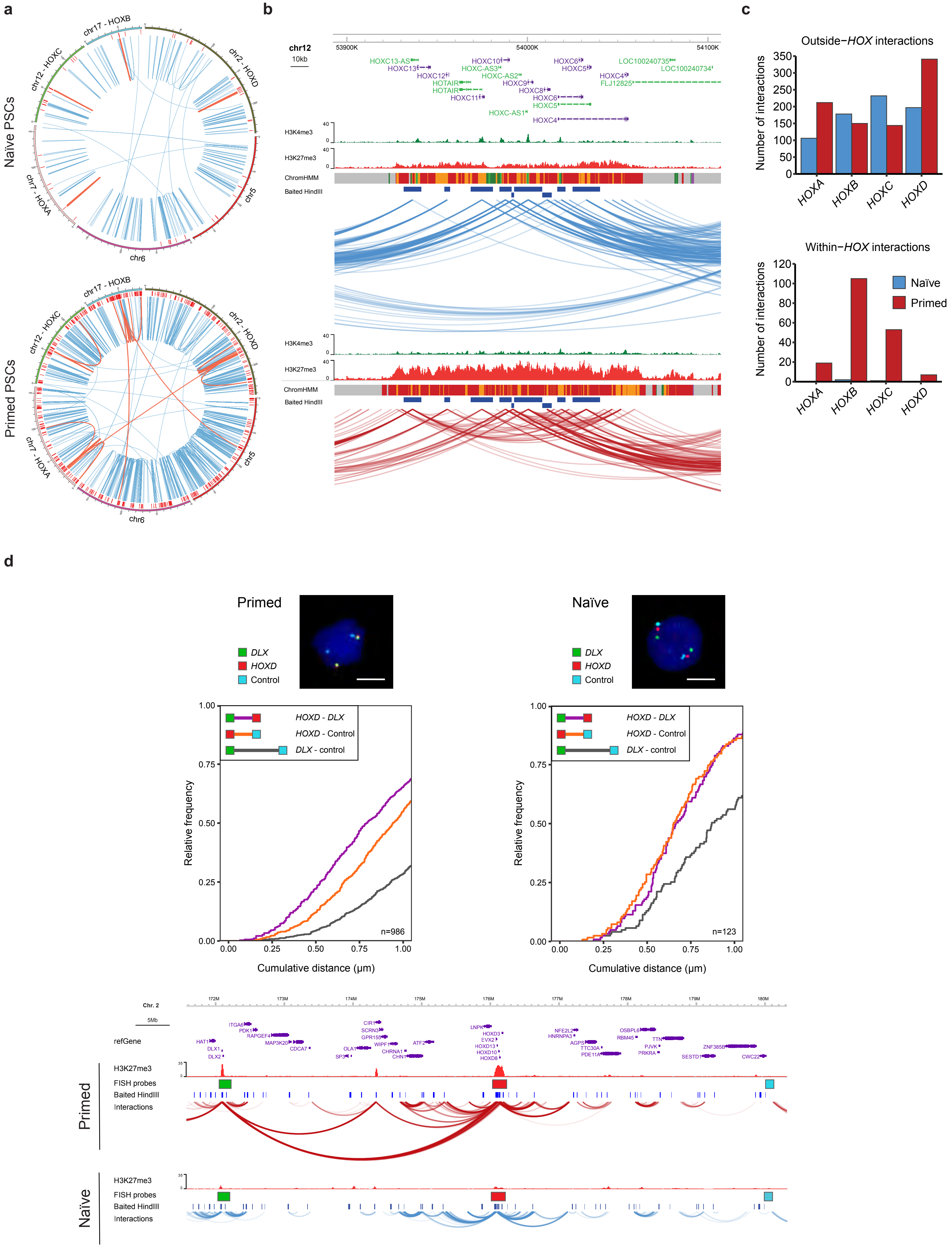

Chovanec Figure S7

Supplementary Fig. 7: Differences in promoter interactions between human pluripotent states

a Circos plots show long-range PCHi-C interactions (blue lines) and interactions with the *HOX* clusters (red lines) for the chromosomes indicated in naive (upper) and primed (lower) PSCs. The red outer track shows H3K27me3-marked regions.

b Genome browser representations of the *HOXC* locus in naive (upper) and primed (lower) PSCs. Tracks shown include all chromatin interactions that have at least one end of an interaction in this region (blue and red lines), baited HindIII fragments, and ChIP-Seq data including H3K4me3, H3K27me3 and ChromHMM states. Note the higher number of interactions within the *HOXC* locus in primed compared to naive PSCs.

c Charts show the number of 'outside' (upper) and 'within' (lower) chromatin interactions for the four *HOX* loci in both cell types. 'Outside' interactions are when one end of the interaction is within the *HOX* region and the other end of the interaction is outside of the region, and 'within' interactions are when both ends of the interaction are within the same *HOX* region. Note the striking difference in the number of 'within' interactions, particularly for the *HOXB* and *HOXC* loci, between pluripotent states.

d Validation of a long-range Polycomb-associated interaction by triple-label 3D DNA FISH in primed (left) and naive (right) PSCs. Representative images show probe signals for *DLX1/2* (green), *HOXD10/11* (red) and control (blue) region. Scale bars, 5µm. Cumulative distribution plots show the relative frequency of alleles colocalising at increasing distance cutoffs; n=986 cells counted for primed and n=123 cells for naive, from one experiment. The results show that in primed PSCs, *HOXD10/11* and *DLX1/2* loci are closer together (purple line) compared to *HOXD10/11* and a control locus that is equidistant in the opposite direction along the chromosome (red line). In contrast, in naive PSCs, there was no difference in the proximity between *HOXD10/11* – *DLX1/2* and *HOXD10/11* – control locus. Genome browser image shows the locations of the three probes used, together with H3K27me3 ChiIP-seq tracks and PCHi-C interactions across this region.

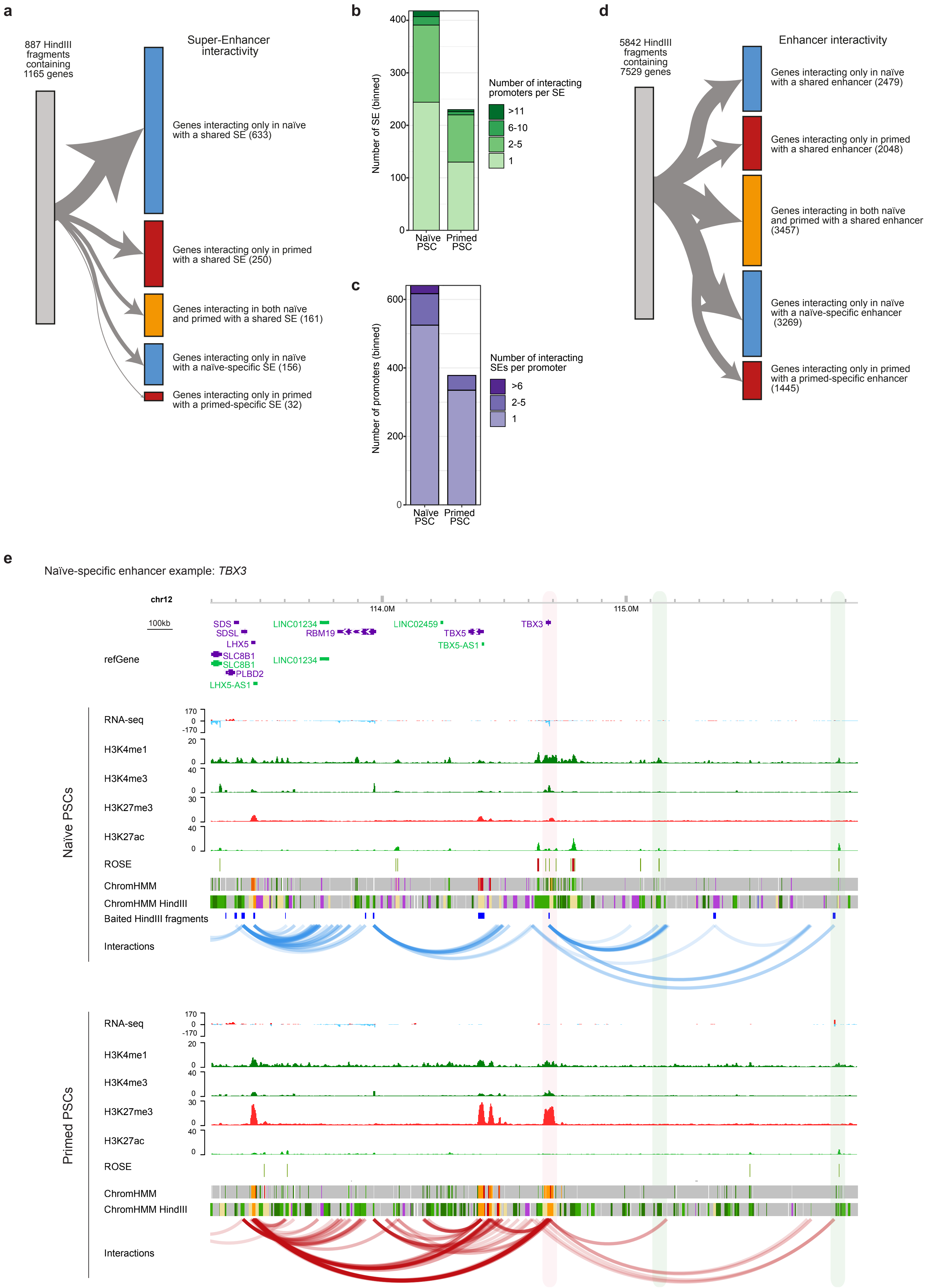

Chovanec Figure S8

Supplementary Fig. 8: Comparing promoter – enhancer interactions between naive and primed PSCs.

a Sankey plot reveals the interactivity patterns for the 887 HindIII regions (containing 1,165 gene promoters) that interact with a SE in either naive or primed PSCs. The right column shows the breakdown of individual regions based on their cell type-specific interactivity and SE classification. The largest category represents gene promoters that interact with a shared SE only in naive PSCs.

b Stacked bar chart showing the binned number of promoters interactions per SE in naive and primed PSCs.

c Stacked bar chart showing the number of SEs interacting with 1, 2–5, or >6 gene promoters in naive and primed PSCs.

d Sankey plot reveals the interactivity patterns for the 5842 HindIII regions (containing 7,529 gene promoters) that interact with an enhancer in either naive or primed PSCs. The right column shows the breakdown of individual regions based on their cell type-specific interactivity and enhancer classification. The largest category represents gene promoters that interact with a shared enhancer in both cell types.

e Genome browser representation shows an example of a naive-specific enhancer at the *TBX3* locus. *TBX3* is highly expressed in naive PSCs, and the *TBX3* promoter (shaded in red box) interacts with distal active enhancers (shaded in green boxes) only in naive PSCs. In primed PSCs, the *TBX3* locus is decorated with H3K27me3, and interacts with distal genes that are also marked by H3K27me3, including *LHX5* and *TBX5*.

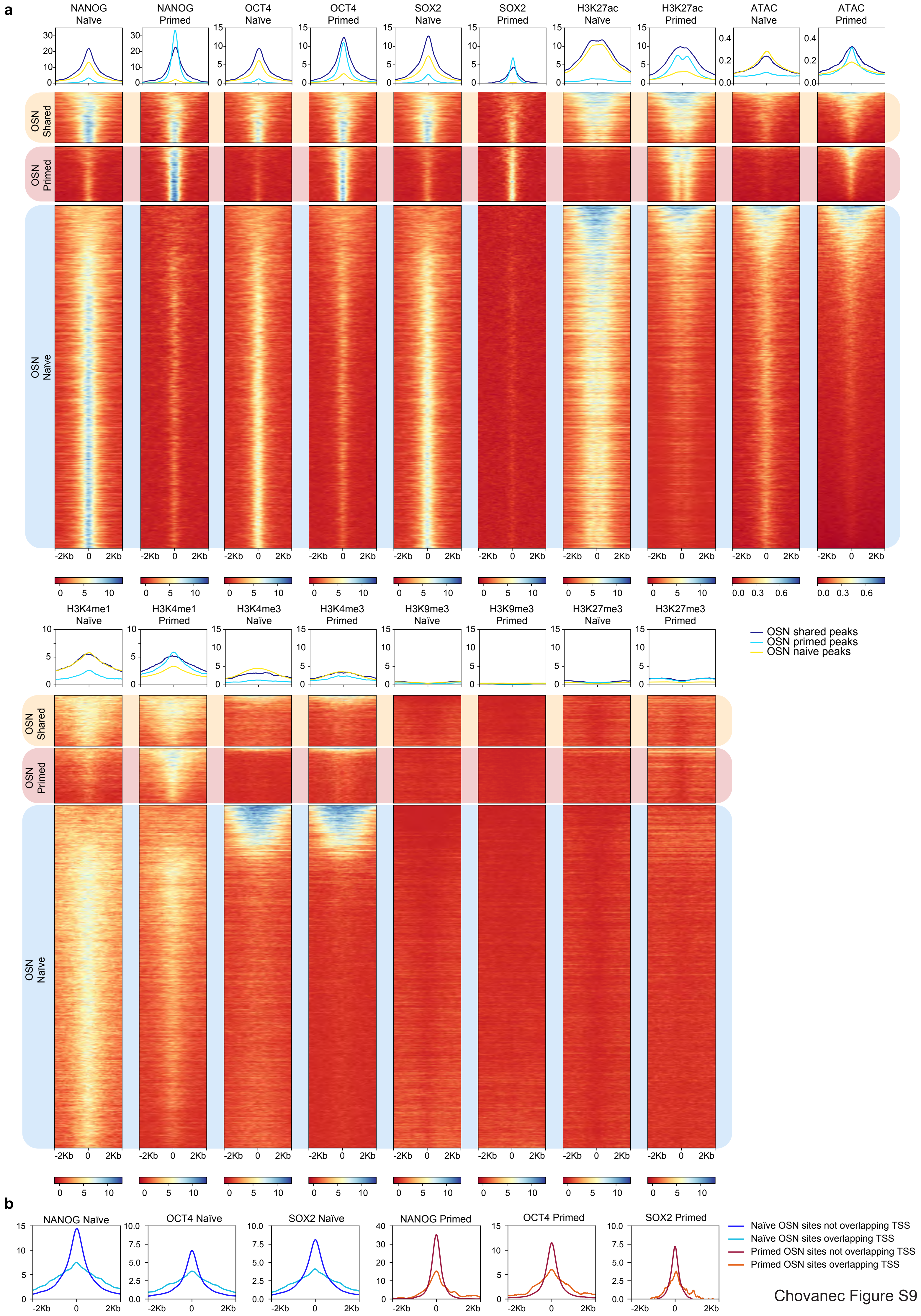

Chovanec Figure S9

Supplementary Fig. 9: Characterisation of OSN sites in naive and primed PSCs

a ChIP-seq and ATAC-seq data for NANOG, OCT4 and SOX2 were used to categorise OSN sites that are specific to either naive PSCs (n=13,462) or primed PSCs (n=2,164) or shared between both pluripotent states (n=1,994). Shown are metaplots (top) and heatmaps (bottom) of log2-transformed read counts within a 4 kb window centred on the OSN peak. Please note that this figure panel is spread over two rows.

b Metaplots show that NANOG, OCT4 and SOX2 ChIP-seq signals are higher at regions that do not overlap with a transcriptional start site compared to regions that do contain a transcriptional start site. Data are log2-transformed read counts within a 4 kb window centered on an OSN peak.

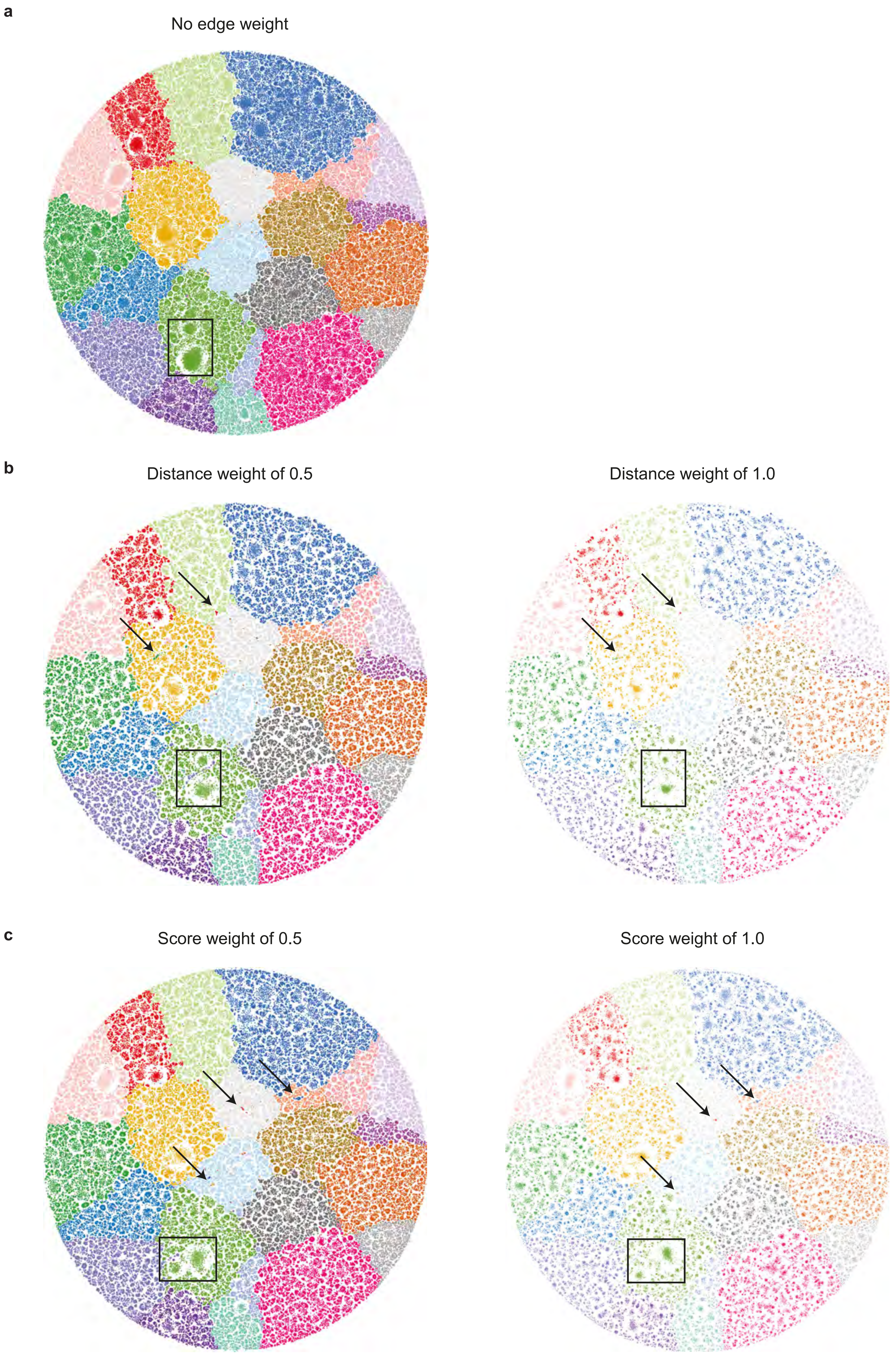

Chovanec Figure S10

Supplementary Fig. 10: Chromosome layout and examination of edge weights on the layout of the PCHi-C network

a Using the circle pack layout implemented in Gephi, it is possible to arrange the network nodes by chromosomes. The subsequent application of the ForceAtlas2 layout results in the network retaining the pre-established chromosome territories.

b We additionally explored the influence of two edge weights on the network layout. Log2 interaction distances were used as edge weights with influence of 0.5 (left) or 1 (right). In the rectangle, the protocadherin gene cluster along with an additional cluster are highlighted. Highlighted with arrows are *trans*-interactions visible as distinct colors within a different chromosome territory.

c CHiCAGO scores were used as edge weights with an influence of 0.5 (left) or 1 (right). An increased edge weight (1 vs 0.5) results in a greater compaction of individual clusters/communities. The edge weights have a minimal influence for the overall layout of the network.

Primed hPSCs

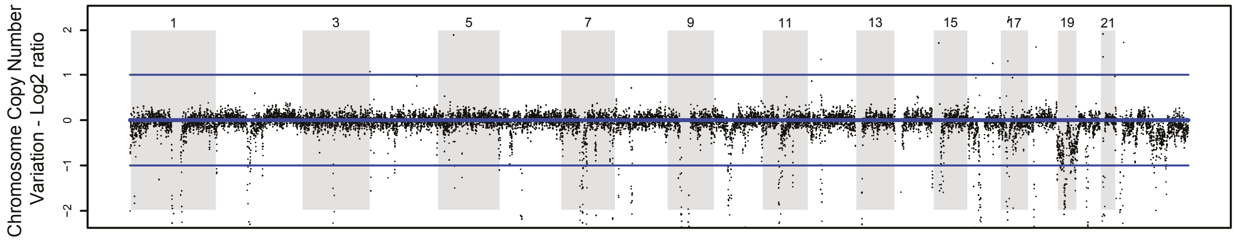

Naïve hPSCs

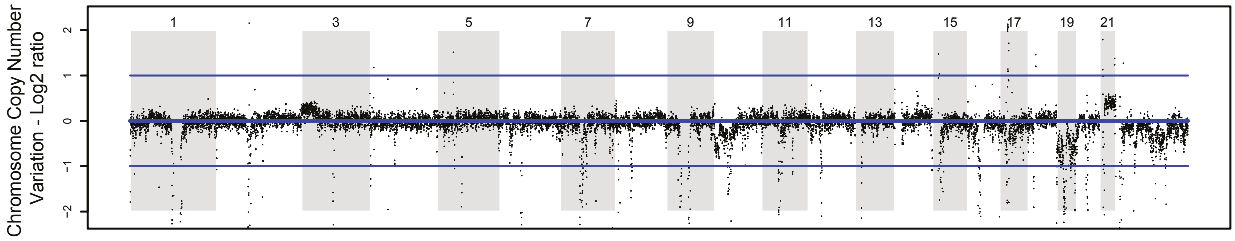

Chovanec Figure S11

Supplementary Fig. 11: Copy number variation analysis of naive and primed PSCs using Hi-C sequencing reads.

Each dot represents a 100kb region. Dots above the center line indicate gain of copy number and below the line indicates loss of copy number. Chromosomes are numbered (above) with alternative white/grey shading.

Supplementary references

Abdennur, N., Mirny, L.A. Cooler: scalable storage for Hi-C data and other genomically labeled arrays. Bioinformatics 36, 311–316 (2020).

Bastian M., Heymann S., Jacomy M. Gephi: an open source software for exploring and manipulating networks. International AAAI Conference on Weblogs and Social Media (2009).

Cairns, J. et al. CHiCAGO: robust detection of DNA looping interactions in Capture Hi-C data. Genome Biol. 17, 127 (2016).

Chen, E.Y. et al. Enrichr: interactive and collaborative HTML5 gene list enrichment analysis tool. BMC Bioinformatics 14, 128 (2013).

Conway, J.R., Lex, A., Gehlenborg, N. UpSetR: An R Package for the Visualization of Intersecting Sets and their Properties. Bioinformatics 33, 2938–2940 (2017).

Csardi, G., Nepusz, T. The igraph software package for complex network research. Complex Systems, 1695 (2006).

da Costa-Luis, C. et al. tqdm v4.19.5. Zenodo (2017).

Durand, N.C. et al. Juicer Provides a One-Click System for Analyzing Loop-Resolution Hi-C Experiments. Cell Syst 3, 95–98 (2016).

Ernst, J., Kellis, M. ChromHMM: automating chromatin-state discovery and characterization. Nat. Methods 9, 215–216 (2012).

Ewels, P. et al. Cluster Flow: A user-friendly bioinformatics workflow tool. F1000Research 5, 2824 (2016).

Flyamer, I.M., Illingworth, R.S., Bickmore, W.A. Coolpup.py: versatile pile-up analysis of Hi-C data. Bioinformatics 36, 2980–2985 (2020).

Gel, B. et al. regioneR: An R/Bioconductor package for the association analysis of genomic regions based on permutation tests. Bioinformatics 32, 289–291 (2016).

Gifford, C.A. et al. Transcriptional and epigenetic dynamics during specification of human embryonic stem cells. Cell 23, 1149-1163 (2013).

Grant, C.E., Bailey, T.L., Noble, W.S. FIMO: scanning for occurrences of a given motif. Bioinformatics 27, 1017–1018 (2011).

Gu, Z., Eils, R., Schlesner, M. Complex heatmaps reveal patterns and correlations in multidimensional genomic data. Bioinformatics 32, 2847-2849 (2016).

Heinz, S. et al. Simple combinations of lineage-determining transcription factors prime cis-regulatory elements required for macrophage and B cell identities. Mol Cell 38, 576-589 (2010).

Huber, W. et al. Orchestrating high-throughput genomic analysis with Bioconductor. Nat. Methods 12, 115–121 (2015).

Hunter, J.D. Matplotlib: A 2D Graphics Environment. Computing in Science & Engineering 9, 90-95 (2007).

Jacomy, M., Venturini, T., Heymann, S., Bastian, M. ForceAtlas2, a continuous graph layout algorithm for handy network visualization designed for the Gephi software. PLoS One 9, e98679 (2014).

Ji, X. et al. 3D chromosome regulatory landscape of human pluripotent cells. Cell Stem Cell 18, 262–275 (2016).

Kim, D., Langmead, B., Salzberg, S.L. HISAT: a fast spliced aligner with low memory requirements. Nat. Methods 12, 357–360 (2015).

Krueger, F., Andrews, S.R. Bismark: a flexible aligner and methylation caller for Bisulfite-Seq applications. Bioinformatics 27, 1571–1572 (2011).

Krzywinski, M.I. et al. Circos: An information aesthetic for comparative genomics. Genome Res. 19, 1639-45 (2009).

Kuleshov, M.V. et al. Enrichr: a comprehensive gene set enrichment analysis web server 2016 update. Nucleic Acids Res. 44, W90–7 (2016).

Langmead, B., Salzberg, S.L. Fast gapped-read alignment with Bowtie 2. Nat. Methods 9, 357–359 (2012).

Lawrence, M. et al. Software for computing and annotating genomic ranges. PLoS Comput. Biol. 9, e1003118 (2013).

Lawrence, M., Gentleman, R., Carey, V. rtracklayer: An R package for interfacing with genome browsers. Bioinformatics 25, 1841-1842 (2009).

Li, H. et al. The Sequence Alignment/Map format and SAMtools. Bioinformatics 25, 2078–2079 (2009).

Love, M. I., Huber, W., Anders, S. Moderated estimation of fold change and dispersion for RNA-seq data with DESeq2. Genome Biol. 15, 550 (2014).

Lovén, J. et al. Selective inhibition of tumor oncogenes by disruption of super-enhancers. Cell 153, 320–334 (2013).

McLeay, R.C., Bailey, T.L. Motif Enrichment Analysis: a unified framework and an evaluation on ChIP data. BMC Bioinformatics 11, 165 (2010).

Pastor, W.A. et al. TFAP2C regulates transcription in human naive pluripotency by opening enhancers. Nat Cell Biol. 20, 553-564 (2018).

Ramírez, F. et al. deepTools2: A next Generation Web Server for Deep-Sequencing Data Analysis. Nucleic Acids Research 44, W160-165 (2016).

Sarkar, D. Lattice: Multivariate Data Visualization with R. Springer (2008).

Takashima, Y. et al. Resetting transcription factor control circuitry toward ground-state pluripotency

in human. Cell 158, 1254–1269 (2014).

Theunissen, T.W. et al. Molecular criteria for defining the naive human pluripotent state. Cell Stem Cell 19, 502-515 (2016).

Theunissen, T.W. et al. Systematic identification of culture conditions for induction and maintenance of naive human pluripotency. Cell Stem Cell 15, 524–526 (2014).

Wang, S. et al. HiNT: a computational method for detecting copy number variations and translocations from Hi-C data. Genome Biol. 21, 73 (2020).

Whyte, W.A. et al. Master transcription factors and mediator establish super-enhancers at key cell identity genes. Cell 153, 307–319 (2013).

Wickham, H. ggplot2: Elegant Graphics for Data Analysis. Springer-Verlag (2016).

Wingett, S. et al. HiCUP: pipeline for mapping and processing Hi-C data. F1000Res. 4, 1310 (2015).

Zhang, Y. et al. Model-based analysis of ChIP-Seq (MACS). Genome Biol. 9, R137 (2008).

Zhou, X. et al. Epigenomic annotation of genetic variants using the Roadmap Epigenome Browser. Nat. Biotechnol. 33, 345–346 (2015).

Zhou, X. et al. Exploring long-range genome interactions using the WashU Epigenome Browser. Nat. Methods 10, 375–376 (2013).

Zhou, X. et al. The Human Epigenome Browser at Washington University. Nat. Methods 8, 989–990 (2011).
