# Supplementary material for: Widespread reorganisation of pluripotent factor binding and gene regulatory interactions between human pluripotent states
Source: Nat Commun. 2021 Apr 7;12:2098. doi: 10.1038/s41467-021-22201-4 (PMC8026613; doi:10.1038/s41467-021-22201-4)
Supplement: Supplementary file 3 — Description of Additional Supplementary Files [file 41467_2021_22201_MOESM3_ESM.pdf]

Supplementary Dataset 1: Annotated list of promoter-interactions in naive and primed PSCs.

Supplementary Dataset 2: GO analysis of genes contained within the Polycomb-associated interaction network. The adjusted *P*-value is the z-score of the deviation from the expected rank by the Fisher exact test as implemented in Enrichr (Chen et al. 2013; Kuleshov et al. 2016).

Supplementary Dataset 3: Definitions used to classify enhancers.

Supplementary Dataset 4: Further information related to the methods.
