# Supplementary material for: Widespread reorganisation of pluripotent factor binding and gene regulatory interactions between human pluripotent states
Source: Nat Commun. 2021 Apr 7;12:2098. doi: 10.1038/s41467-021-22201-4 (PMC8026613; doi:10.1038/s41467-021-22201-4)
Supplement: Supplementary file 8 — Reporting Summary [file 41467_2021_22201_MOESM8_ESM.pdf]

nature research

Corresponding author(s): Peter Rugg-Gunn

Last updated by author(s): Jan 16, 2021

Reporting Summary

Nature Research wishes to improve the reproducibility of the work that we publish. This form provides structure for consistency and transparency in reporting. For further information on Nature Research policies, see our Editorial Policies and the Editorial Policy Checklist.

Statistics

For all statistical analyses, confirm that the following items are present in the figure legend, table legend, main text, or Methods section.

n/a	Confirmed	
☐	☒	The exact sample size (*n*) for each experimental group/condition, given as a discrete number and unit of measurement
☒	☐	A statement on whether measurements were taken from distinct samples or whether the same sample was measured repeatedly
☐	☒	The statistical test(s) used AND whether they are one- or two-sided<br>*Only common tests should be described solely by name; describe more complex techniques in the Methods section.*
☒	☐	A description of all covariates tested
☐	☒	A description of any assumptions or corrections, such as tests of normality and adjustment for multiple comparisons
☐	☒	A full description of the statistical parameters including central tendency (e.g. means) or other basic estimates (e.g. regression coefficient) AND variation (e.g. standard deviation) or associated estimates of uncertainty (e.g. confidence intervals)
☐	☒	For null hypothesis testing, the test statistic (e.g. *F*, *t*, *r*) with confidence intervals, effect sizes, degrees of freedom and *P* value noted<br>*Give P values as exact values whenever suitable.*
☒	☐	For Bayesian analysis, information on the choice of priors and Markov chain Monte Carlo settings
☒	☐	For hierarchical and complex designs, identification of the appropriate level for tests and full reporting of outcomes
☒	☐	Estimates of effect sizes (e.g. Cohen's *d*, Pearson's *r*), indicating how they were calculated

Our web collection on statistics for biologists contains articles on many of the points above.

Software and code

Policy information about availability of computer code

Data collection

Illumina HiSeq 2500 Control Software v2.2.58. Illumina HiSeq 2500 RTA v1.18.64.

Data analysis

Trim Galore v0.4.5 Babraham Bioinformatics https://www.bioinformatics.babraham.ac.uk/projects/trim_galore/
Bismark v0.18.2 Krueger at al. 2011 https://www.bioinformatics.babraham.ac.uk/projects/bismark/
Bowtie2 v2.2.7 & v2.3.2 Langmead et al. 2012 http://bowtie-bio.sourceforge.net/bowtie2/index.shtml
SeqMonk v1.45.3 Babraham Bioinformatics https://www.bioinformatics.babraham.ac.uk/projects/seqmonk/
Cluster Flow v0.5 Ewels et al. 2016 https://clusterflow.io
R v3.6 – 4.0 R Core Team https://www.R-project.org/
igraph v1.2.1 Csardi and Nepusz 2006 https://igraph.org
regioneR v1.10.0 Gel et al. 2016 http://bioconductor.org/packages/release/bioc/html/regioneR.html
ComplexHeatmap v1.17.1 Gu et al. 2016 https://www.bioconductor.org/packages/release/bioc/html/ComplexHeatmap.html
data.table v1.10.4-3 Matt Dowle https://rdatatable.gitlab.io
DESeq2 v1.18.1 Love et al. 2014 https://bioconductor.org/packages/release/bioc/html/DESeq2.html
GenomicFeatures and GenomicRanges v1.30.3 Lawrence et al. 2013 https://bioconductor.org/packages/release/bioc/html/GenomicFeatures.html
ggpubr v0.1.6 Alboukadel Kassambara https://rpkgs.datanovia.com/ggpubr/
pheatmap v1.0.8 Raivo Kolde https://github.com/raivokolde/pheatmap
plyr v1.8.4 Hadley Wickham http://had.co.nz/plyr/
dplyr v0.7.8 Hadley Wickham https://dplyr.tidyverse.org
tidyr v0.8.0 Hadley Wickham https://tidyr.tidyverse.org
naniar v0.4.1 Nicholas Tierney https://github.com/njtierney/naniar
rtracklayer v1.38.3 Lawrence et al. 2009 https://www.bioconductor.org/packages/release/bioc/html/rtracklayer.html
BSgenome.Hsapiens.UCSC.hg38 The Bioconductor Dev Team https://bioconductor.org/packages/release/data/annotation/html/

BSgenome.Hsapiens.UCSC.hg38.html
Bioconductor Huber et al. 2015 & Gentleman et al. 2004 https://www.bioconductor.org
UpSetR v1.3.3 Conway et al. 2017 http://gehlenborglab.org/research/projects/upsetr/
venneuler v1.1-0 Lee Wilkinson http://www.rforge.net/venneuler/
sushi v1.16.0 Douglas H Phanstiel https://bioconductor.org/packages/release/bioc/html/Sushi.html
ggplot2 v3.1.0.9000 Wickham 2016 https://ggplot2.tidyverse.org
ggrastr v0.1.6 Viktor Petukhov https://github.com/VPetukhov/ggrastr
ggforce v0.2.2 Thomas Lin Pedersen https://ggforce.data-imaginist.com
Lattice v0.20-38 Sarkar 2008 http://lattice.r-forge.r-project.org/index.php
Gmisc v1.6.1 Max Gordon https://cran.r-project.org/web/packages/Gmisc
Cowplot v0.9.2 Claus O. Wilke https://wilkelab.org/cowplot/
Reshape2 v0.8.7 Hadley Wickham https://github.com/hadley/reshape
Circos v0.69-5 Krzywinski et al. 2009 http://circos.ca/
Enrichr Chen et al. 2013 & Kuleshov et al. 2016 https://amp.pharm.mssm.edu/Enrichr/
ROSE Whyte et al. 2013 & Loven et al. 2013 http://younglab.wi.mit.edu/super_enhancer_code.html
WashU Epigenome Browser v48.2.0+ Zhou et al. 2011, 2013, 2015 https://epigenomegateway.wustl.edu
Samtools v1.7 Li et al. 2009 http://www.htslib.org
MACS2 v2.1.1.20160309 Zhang et al. 2008 http://liulab.dfci.harvard.edu/MACS/
fastqc v0.11.7 Babraham Bioinformatics https://www.bioinformatics.babraham.ac.uk/projects/fastqc/
Python 3.5 Python Software Foundation https://www.python.org
Pandas v0.22.0 McKinney 2017 https://pandas.pydata.org
Numpy v1.15.0 https://numpy.org
Bx-python v 0.7.3 James Taylor https://github.com/bxlab/bx-python
Tqdm v4.19.5 da Costa-Luis 2019 https://github.com/tqdm/tqdm
Matplotlib v2.1.2 Hunter 2007 https://matplotlib.org/index.html
Docker for 4DN Hi-C processing pipeline v42 4D Nucleome Network https://github.com/4dn-dcic/docker-4dn-hic
Gephi v0.9.2 Bastian et al. 2009 https://gephi.org
AME v5.0.5 McLeay and Bailey 2010 http://bioinformatics.org.au/ame
FIMO Grant at el. 2011 http://meme-suite.org
HiCUP v0.5.8 Wingett et al. 2015 https://www.bioinformatics.babraham.ac.uk/projects/hicup/
HOMER v4.7 Heinz et al. 2010 http://homer.ucsd.edu/homer/
Juicer tools v1.8.9 Durand et al. 2016 https://github.com/aidenlab/juicer
Juicebox v1.8.8 Durand et al. 2016 https://github.com/aidenlab/Juicebox
CHiCAGO Cairns et al. 2016 https://bitbucket.org/chicagoTeam/chicago
Deeptools v3.1.0 Ramirez et al. 2016 https://deeptools.readthedocs.io/en/develop/
HiNT-CNV tool Wang et al. 2020 HiNT-CNV tool
Cooler Abdennur and Mirny https://github.com/mirnylab/cooler
Metafer v4.0 https://metasystems-international.com/us/products/metafer/
ForceAtlas2 Jacomy et al. 2014. https://doi.org/10.1371/journal.pone.0098679

For manuscripts utilizing custom algorithms or software that are central to the research but not yet described in published literature, software must be made available to editors and reviewers. We strongly encourage code deposition in a community repository (e.g. GitHub). See the Nature Research guidelines for submitting code & software for further information.

Data

Policy information about availability of data

All manuscripts must include a data availability statement. This statement should provide the following information, where applicable:

- Accession codes, unique identifiers, or web links for publicly available datasets
- A list of figures that have associated raw data
- A description of any restrictions on data availability

The Hi-C, PCHi-C, ChIP-Seq and CUT&RUN data reported in this paper have been deposited at the Gene Expression Omnibus under accession number GSE133126. The Reviewer access token is provided in the manuscript. Processed data including CHiCAGO objects containing all detected interactions, WashU Genome Browser tracks, and TAD and compartment information have been made available through the Open Science Framework (https://osf.io/jp29m).

Field-specific reporting

Please select the one below that is the best fit for your research. If you are not sure, read the appropriate sections before making your selection.

☒ Life sciences ☐ Behavioural & social sciences ☐ Ecological, evolutionary & environmental sciences

For a reference copy of the document with all sections, see nature.com/documents/nr-reporting-summary-flat.pdf

Life sciences study design

All studies must disclose on these points even when the disclosure is negative.

Sample size	No sample size calculations were performed. Sample sizes were chosen to allow sufficient statistical analysis to be performed. The recommended number of replicates required for packages such as DESeq2, CHiCAGO, and MACS2 were used.
Data exclusions	No data were excluded from the analyses.

Replication	All replication attempts were successful and well correlated. Independent biological duplicate experiments were performed for PCHiC, HiC, ChIP-seq, and CUT&RUN experiments.
Randomization	Samples were not randomised and were allocated into experimental groups by cell type.
Blinding	Investigators were not blinded to the sample identity as all data produced was from objective quantitative methods and so subjective bias was not relevant.

Reporting for specific materials, systems and methods

We require information from authors about some types of materials, experimental systems and methods used in many studies. Here, indicate whether each material, system or method listed is relevant to your study. If you are not sure if a list item applies to your research, read the appropriate section before selecting a response.

Materials & experimental systems

n/a	Involved in the study
☐	☒ Antibodies
☐	☒ Eukaryotic cell lines
☒	☐ Palaeontology and archaeology
☒	☐ Animals and other organisms
☒	☐ Human research participants
☒	☐ Clinical data
☒	☐ Dual use research of concern

Methods

n/a	Involved in the study
☐	☒ ChIP-seq
☒	☐ Flow cytometry
☒	☐ MRI-based neuroimaging

Antibodies

Antibodies used	H3K4me1 Abcam ab8895 - 5µg antibody per IP<br>NANOG R&D AF1997 - 5µg antibody per IP<br>SOX2 R&D AF2018 - 5µg antibody per IP<br>IgG Jackson ImmunoResearch 315-005-003 - 5µg antibody per IP
Validation	H3K4me1 Abcam ab8895 - has been validated in human cells for the application of ChIP<br>NANOG R&D AF1997 -has been validated in human cells for the application of ChIP<br>SOX2 R&D AF2018 - has been validated in human cells for the application of ChIP<br>IgG Jackson ImmunoResearch 315-005-003 - has been validated in human cells for the application of ChIP<br>Additional validation statements for other species and applications are found on the manufacturer's product pages.

Eukaryotic cell lines

Policy information about cell lines

Cell line source(s)	WA09/H9 NK2 naïve and primed PSCs were obtained from the laboratory of Austin Smith with the permission of WiCell.
Authentication	Cell lines were authenticated by transcript and protein expression analysis (Takashima et al. 2014; Collier et al. 2017), and G-banded karyotypes (Takashima et al. 2014).
Mycoplasma contamination	All cell lines tested negative for mycoplasma.
Commonly misidentified lines (See ICLAC register)	None of the cell lines used in this study are on the register for commonly misidentified lines.

ChIP-seq

Data deposition

☒ Confirm that both raw and final processed data have been deposited in a public database such as GEO.

☒ Confirm that you have deposited or provided access to graph files (e.g. BED files) for the called peaks.

Data access links<br>*May remain private before publication.*	The Hi-C, PCHi-C, ChIP-Seq and CUT&RUN data reported in this paper have been deposited at GEO under accession number GSE133126.
Files in database submission	Hi-C:<br><br>GSM3900541 HiC_hESC_naive<br>GSM3900542 HiC_hESC_primed

PCHi-C:

GSM3900543 PARC_naive_hESC_rep1
GSM3900544 PARC_naive_hESC_rep2
GSM3900545 PARC_primed_hESC_rep1
GSM3900546 PARC_primed_hESC_rep2

ChIP-seq:

GSM3900535 Input_naive_rep1
GSM3900536 Input_naive_rep2
GSM3900537 NANOG_naive_rep1
GSM3900538 NANOG_naive_rep2
GSM3900539 SOX2_naive_rep1
GSM3900540 SOX2_naive_rep2

CUT&RUN:
GSM4798820 H3K4me1_naive_rep1
GSM4798821 H3K4me1_naive_rep2
GSM4798822 H3K4me1_primed_rep1
GSM4798823 H3K4me1_primed_rep2
GSM4798824 H3K4me1_primed_rep3

Genome browser session
(e.g. UCSC)

The OSF project contains WashU genome browser tracks including of PCHi-C, ChIP-Seq and CUT&RUN.

Methodology

Replicates

Hi-C: One biological replicate for each cell type.

PCHi-C: Two biological replicates per cell type.

ChIP-seq: Two biological replicates provided for SOX2, NANOG and input in naive human pluripotent stem cells.

CUT&RUN: Two biological replicates provided for H3K4me1 in naive cells and three biological replicates provided in primed cells.

Sequencing depth

Hi-C and PCHi-C: 50bp paired-end reads. >190 million total reads per sample.

ChIP-seq: 75bp single-end reads. Each sample was sequenced to a depth of >17million total reads per sample.

CUT&RUN: 75bp paired-end reads. Each sample was sequenced to a depth of >7million total reads per sample.

Antibodies

H3K4me1 Abcam ab8895
NANOG R&D AF1997
SOX2 R&D AF2018
IgG Jackson ImmunoResearch 315-005-003

Peak calling parameters

Hi-C
HiCUP was used to map and filter di-tags to human genome build GRCh38. The aligned Hi-C data were normalized using HOMER v4.7 and Juicer tools v1.8.9. Using binned Hi-C data, we computed the coverage- and distance-related background in the Hi-C data employing matrix balancing algorithms at 25 kb and 250 kb (iterative correction by HOMER) and a 5 kb to 2.5 Mb (Knight–Ruiz balancing by Juicer tools) range of resolutions. We compared global organisation by plotting the log10 frequency of cis-chromosomal contacts in the raw data at various genomic distances on the log10 scale. TADs were identified based on directionality indices of Hi-C interactions, using HOMER with minDelta=2 and other parameters kept at their default values. This resulted in 3,124 TADs for naive PSCs and 2,917 TADs for primed PSCs. Hi-C peaks were identified using HiCCUPS v1.8.8 with the following parameters: --ignore_sparsity -k KR -f 0.1 -r 250000 -d 750000 -i 8 -p 4. The peaks identified on chromosome 5 in primed PSCs were used for both naive and primed aggregate peak analysis with the following parameters: -r 250000 -c chr5 -n 30 -w 10 using Juicer tools v1.8.9.

PCHi-C
PCHi-C data were mapped and filtered using HiCUP with the GCRh38 human genome build. CHiCAGO was used to define significant promoter interactions at the level of individual HindIII fragments. Two biological replicates for each cell type were normalised and combined as part of the CHiCAGO pipeline. CHiCAGO interaction scores correspond to –log-transformed, weighted p-values for each fragment read pair. A CHiCAGO interaction score of 5 or above was considered significant based on previous empirical observations.

ChIP-seq and CUT&RUN:
Reads were trimmed using Trim Galore and mapped to human genome GRCh38 using Bowtie2. All analyses were performed using SeqMonk and R. Peaks were called using MACS2 with parameters q<10-9 for all histone modification samples except for H3K4me1 for which the cutoff used was q<10-7. For quantitation, read lengths were extended to 300 bp and regions of coverage outliers were excluded. OCT4, NANOG and SOX2 peaks were called using a SeqMonk implementation of MACS with parameters p<10-5, sonicated fragment size = 300. Peaks were filtered by signal intensity, retaining only peaks that overlap with at least one 500 bp window in which log2 RPM > 0. Regions of OCT4, NANOG and SOX2 peaks were combined and merged if closer than 100 bp. The resulting list of regions was filtered for those that overlap with MACS peaks for all three factors and called OSN peaks. Control regions are 10000 randomly selected 1.2 kb windows (approximate average peak size). TFAP2C peaks were called using a SeqMonk implementation of MACS with parameters p<10-5, sonicated fragment size = 300 for individual replicates, and the overlap between replicates of the resulting regions were used for quantitation.

Data quality	This information is provided in the 'Peak calling parameters' section above.
Software	This information is provided in the 'Peak calling parameters' section above.
